# Sustained Functioning Impairments and Oxidative Stress with Neurobehavioral Dysfunction Associated with Oral Nicotine Exposure in the Brain of a Murine Model of Ehrlich Ascites Carcinoma: Modifying the Antioxidant Role of *Chlorella vulgaris*

**DOI:** 10.3390/biology11020279

**Published:** 2022-02-10

**Authors:** Amany Abdel-Rahman Mohamed, Khlood M. El Bohy, Gihan G. Moustafa, Hesham H. Mohammed, Mohamed M. M. Metwally, Heba El Desoukey Mohammed, Mohamed A. Nassan, Taghred M. Saber

**Affiliations:** 1 Department of Forensic Medicine and Toxicology, Faculty of Veterinary Medicine, Zagazig University, Zagazig 4511, Egypt; kholoode@zu.edu.eg (K.M.E.B.); gihanmm@zu.edu.eg (G.G.M.); taghredahmed@zu.edu.eg (T.M.S.); 2 Department of Veterinary Public Health, Faculty of Veterinary Medicine, Zagazig University, Zagazig 44519, Egypt; heshamaa@zu.edu.eg; 3 Department of Pathology, Faculty of Veterinary Medicine, Zagazig University, Zagazig 44511, Egypt; mmetwally@zu.edu.eg; 4 Specialist of Forensic Medicine and Toxicology, Veterinary Services, El Senbellawein 35931, Egypt; hebomm@zu.edu.eg; 5 Department of Clinical Laboratory Sciences, Turabah University College, Taif University, P.O. Box 11099, Taif 21944, Saudi Arabia; m.nassan@tu.edu.sa

**Keywords:** Nicotine, *Chlorella vulgaris*, cancer, apoptosis, neurobehavior, DNA damage, histopathology, immunohistochemistry

## Abstract

**Simple Summary:**

Nicotine is the major psychoactive component considered to underlie tobacco’s addictive nature, and its dependence has been linked to several drawbacks on behavior and brain health. The purpose of this study was to investigate the mechanisms triggered by oral nicotine that cause brain tissue damage, as well as the supportive role of *Chlorella vulgaris* microalgae supplementation in Ehrlich ascites carcinoma in mice. The results revealed pronounced neurobehavioral alterations, increased mortality rate, oxidative stress, DNA damage, and augmented inflammatory response in the brain tissue alongside the microstructural alteration caused by nicotine. *Chlorella vulgaris* was quite successful in reducing the negative effects of nicotine. It acts as an antioxidant anti-inflammatory and restores nearly normal tissue architectures. As a result, we believe it should be supplemented to cancer patients consuming regular nicotine doses.

**Abstract:**

*Background*: This study provides a model for studying the mechanism(s) responsible for the nervous tissue damage and misfunctioning that occurred due to oral nicotine exposure, considered a stress factor, during the presence of Ehrlich ascites carcinoma bearing in the mouse model (EAC). The mitigating role of *Chlorella vulgaris (CV*) against nicotine-induced brain damage was evaluated. *Methods*: Eighty Swiss female mice were classified into four groups, these were the control, the *CV* group, the nicotine group(100 µg/kg), and the combination group. Oxidant/antioxidant status, proinflammatory cytokines levels, DNA damage, quantitative microscopical lesions, and Caspase 3, Bcl-2 proteins were assessed in the current study. Levels of dopamine (DA) and gamma-aminobutyric acid (GABA) were also evaluated. *Results*: Nicotine was found to cause pronounced neurobehavioral alterations, increase the mortalities oxidative stress DNA damage, and augment the inflammatory response in brain tissue alongside the microstructural alteration. The administration of *CV* with nicotine in EAC-bearing mice rescued the detrimental effects of nicotine. *Conclusions*: *CV* aids in reducing the harmful effects of nicotine and returns the conditions caused by nicotine to near-control levels. Thus, we are in favor of giving it to cancer patients who are taking daily dosages of nicotine even by smoking cigarettes or being exposed to second-hand smoke.

## 1. Introduction

The tobacco epidemic is one of the world’s major public health hazards, killing more than 8 million people each year. Moreover, 7 million of those deaths are directly related to tobacco use, whereas around 1.2 million are related to nonsmokers being exposed to second-hand smoke [1]. Although around 4000 compounds occur in cigarette smoke, nicotine is the most dynamic alkaloid in tobacco [2]. Nicotine is the major psychoactive component considered to underlie tobacco’s addictive nature. In addition to its high addiction potential, nicotine dependence has been linked to an unusually high number of neuropsychiatric illnesses, such as schizophrenia, anxiety and mood disorders, and co-dependency on other substances, including alcohol and cannabis [3,4].

Nicotine works by releasing dopamine into the body’s neurological system via a molecular mechanism. Nicotine dependency and withdrawal symptoms develop after repeated exposure. Anyone who has tried to stop using tobacco products would recognize this response. Scientists are continue to reveal the negative effects of nicotine beyond its addictiveness. Numerous acute and long-term consequences of nicotine on the brain have been clarified through decades of pre-clinical neuroscience study. Furthermore, nicotine has been shown to influence a number of neurotransmitter systems implicated in mood and anxiety control, including GABA (glutamate), dopamine (DA), and serotonin (5-HT). Precise neurodevelopmental pathways must functionally regulate these neurotransmitter systems to work correctly in the long run. Moreover, alterations in these neuropharmacological components and the accompanying molecular signaling reaction cascades may act as primary critical biomarkers for various neuropsychiatric illnesses, including mood disorders and anxiety ailments. In addition to these several changes, for many years, doctors have known that smoking is the leading cause of lung cancer. It is still true today that smoking cigarettes or being exposed to second-hand smoke causes roughly nine out of 10 lung cancer fatalities [5]. Nicotine initiates a process known as epithelial-mesenchymal transition, a critical step to malignant cell development [6]. Nicotine lowers the tumor suppressor gene CHK2. This may allow nicotine to bypass one of the body’s natural cancer defenses [7]. In different societies, we may find many cancer patients who are smokers and still smoke even after discovering their disease. On the other hand, they may be passive smokers or what is called second-hand smokers, regardless of the type and cause of cancer, the condition, and whether nicotine is the cause of cancer or not. 

Currently, analysis of changes in neurotransmitter extracellular levels in distinct brain areas is an important method for identifying the neuronal systems participating in specific activities related to the nervous system and the rate of activity. Neurotransmitters like acetylcholine (ACh), glutamate, and -aminobutyric acid (GABA) have been studied in a variety of cases, including models related to memory, and there has been considerable evidence of variation in extracellular levels that correlate with changes in neuronal activity, particularly when a person is exposed to nicotine or consumes cigarettes.

Ehrlich ascites carcinoma (EAC) is an undifferentiated carcinoma with rapid growth, high transplantability, and a short life span [8]. EAC bears closeness to human tumors; consequently, the ascetic forms of this tumor are commonly used to study the cancer model and assume the effectiveness of different natural products to overcome the side effects of other environmental agents [9].

In this era, there is a growing interest in using natural items for medicinal purposes or to mitigate pharmacological side effects [10,11,12,13]. Because of its potential health benefits, studies involving the usage of microalgae have sparked a lot of interest. Chlorella is a green unicellular alga that is produced for commercial purposes and marketed as a nutritional supplement all over the world [14,15]. The study of Chlorella’s dietary implication in human health began in the early 1950’s when Chlorella as a food source was initiated during a global food crisis [16]. Chlorella produces lutein, a chemical that has been shown to thwart and treat macular degeneration and has anti-cataract characteristics [17,18]. Chlorella extracts have been shown to have antitumor [19,20], anti-inflammatory [21], and antioxidant [22] properties. Chlorella has been shown to reduce cholesterol concentrations, regulate blood pressure, accelerate wound healing, and boost the immune system [23]. It can also alleviate symptoms and enhance the quality of life for patients with ulcerative colitis [24], fibromyalgia, and hypertension [25,26]. Chlorella suppressed aortic atheromatous lesions and significantly reduced low-density lipoprotein (LDL) cholesterol levels [27].

Although many studies discussed the potential supplementation of *Chlorella vulgaris (CV*) as a food supplement [15,25,28], scientific evidence does not support its efficacy in preventing or treating any disease or supporting toxicity in humans. Because *CV* includes a variety of minerals, antioxidant vitamins such as D and B12, and folates absent in plant-derived food sources [14], it is hypothesized to scavenge free radicals produced by environmental factors such as smoking, meaning that it has antioxidative properties. We also hypothesized that it could be a neurobehavioral protectant. Furthermore, there was very little available literature on the consequences of oral nicotine administration on the health and function of the brain in cancer patients. Hence, in the current study, we intended to investigate if *CV* could be an active candidate to protect against the side effects of nicotine on brain function, microstructural lesions, antioxidants, DNA damaging effects, and behavioral alterations in a murine model of Ehrlich ascites carcinoma (EAC) and nicotine exposure. Consequently, we used, for the first time, an in vivo model of female mice to test this hypothesis and mimic the real exposure scenarios. We attempted to connect the changes in neurobehavioral performance that occur as a result of nicotine consumption with the levels of neurotransmitters and observable lesions that are associated with nicotine consumption.

## 2. Materials and Methods

### 2.1. Tested Substances and Chemicals

Nicotine (C10H14N2), of analytical grade, was purchased from Lab Chemicals Trading Co. (Al Eini, El-Sayeda Zainab, Cairo, Egypt) and produced by Sigma-Aldrich (Sigma, St Louis, MO, USA). The *CV* powder was obtained from the Feed Crop Department, Crop Research Institute, Agricultural Research Center, Giza, Egypt.

### 2.2. Quantitative Assessment of Different Components of Chlorella vulgaris by HPLC

The Preparation of Alcoholic Extract (80%): As soon as the *C. vulgaris* powdered leaf material had been air dried, it was extracted with 80% ethanol and heated under reflux for an extended period of time. After that, it was filtered, concentrated, and stored in an airtight container according to the method described by Elsawi et al. [29]. The HPLC technique was applied according to the in-house method of the chromatography lab in the National Research Centre, Dokki, Giza, Egypt. The analysis of phenolic compounds was performed by HPLC-(Agilent 1100, Merck KGaA, Darmstadt, Germany), which is composed of two LC-pumps, a UV/Vis detector, and a C18 column (125 mm × 4.60 mm, 5 µm particle size). Chromatograms were obtained and analyzed using the Agilent ChemStation Phenolic acids and were then separated by employing a mobile gradient phase of two solvents was used—Solvent A (Methanol) and Solvent B (Acetic acid in water (1:25).

For flavonoids, the gradient program started with 100% B and was held at this concentration for the first 3 min as mentioned before. This was followed by 50% eluent A for the next 5 min, after which the concentration of A was increased to 80% for the next 2 min and then reduced to 50% again for the following 5 min detection wavelength at 250 nm. Flavonoids Analyses were performed by HPLC-(Agilent 1100), which is composed of two LC- pumps, a UV/Vis detector, and a C18 column (250 mm × 4.6 mm, 5 µm). The mobile phase consisted of acetonitrile(Sigma, St Louis, MO, USA) (A) and 0.2% (*v*/*v*) aqueous formic acid(Sigma, St Louis, MO, USA) (B) with an Isocratic elution (70:30) program. The detection wavelength was set at 360 nm.The HPLC Isocratic separation of polysaccharides was applied via a C18 reversed-phase column (150 mm × 4.6 mm i.d.; 5 µm) and the mobile phase (Acetonitrile/2%H_3_PO_4_ *v*/*v* 40/60) separations were performed at room temperature with a flow rate of 1 mL/min and an injection loop of 20 µL. Quantitative analysis was performed at 254 nm.

### 2.3. Animals

Eighty adult female Swiss albino mice (average weight 20–22 g) were received from the veterinary medicine laboratory animal farm at Zagazig University in Egypt. All mice were raised under stringent hygienic standards in an atmosphere free of pathogens at 60% relative humidity and 21–24 °C. Mice were fed a healthy balanced diet and had ad libitum access to food and water, except during the exposure and testing periods. They were left for arounf 14 days to acclimatize the lab. All research procedures were carried out under the National Institutes of Health guidelines for the Care and Use of Laboratory Animals and were approved by the Ethics of Animal Use in Research Committee (IACUC), Zagazig University, Egypt, under the reference number (ZU-IACUC/2/F/61/2021).

### 2.4. Experimental Design

The mice were divided randomly into four different treatment groups in which the animals received different drinking solutions via feeding needles. We prepared a solution of 2% saccharin solution (Sigma-Aldrich; Sigma, St Louis, MO, USA) in tap water. This is used as a vehicle for nicotine as it increases the palatability of the solution, which contains the very bitter taste of nicotine that may alter the food and water intake of the mice and affect the behavior of tested animals. The groups of the current experiment were organized as the following:

Group I (control): received 0.1 mL of 2% saccharin solution.

Group II (Nicotine): received 100 µg/mL/kg BW (free base; Sigma) nicotine in 2% saccharin solution [30].

Group III (*CV*): received the *CV* powder (100 mg/kg [31]) dissolved in distilled water at a dose of.

Group IV (Nicotine + *CV*): received a combination of nicotine and *CV* using the pattern mentioned above. All the treatments were continued for 28 days (5 days a week) before tumor induction and continued for 12 days in tumor-bearing mice.

The rationale for choosing this dose of nicotine is based on the chronic oral nicotine exposure in female C57Bl/6 mice, in which the authors used three doses of nicotine (50, 100 and 200 µg/mL/kg [30,32] free base) in drinking water. The mice that received oral nicotine had concentration-related elevation in their plasma cotinine levels (the major nicotine metabolite), equivalent to the nicotine intake, while the high dose resulted in reduced feed and water intake. This intake of nicotine could result in biochemical and behavioral alterations that may be similar to changes that arise following other routes of chronic nicotine delivery even by intravenous or inhalation routes. So, in the current study, we have chosen to test the effects of the middle dose on the different areas of brain health, structure, and function and to estimate the correlation of this dose with brain lesions and also the *CV*’s ability to mitigate such dose effects. This dose is also considered to be about 1/30 of the LD50 of mice, which is estimated by 3 mg/kg BW. Herein, the dose presented in the present study regarding *CV* was tested before for its ability to exert antitumor properties and prolong the survival of mice inoculated with EAC [31], so we tested its ability to exacerbate the neurotoxic impact of nicotine exposure. 

### 2.5. Ehrlich Ascites Carcinoma Cells

The National Cancer Institute of Cairo University, Egypt, kindly provided the parent line of EAC cells. The cell lines were examined for viability [33]. The tumor line was sustained by serial intraperitoneal transplantation of Ehrlich ascites carcinoma 2.5 × 10^6^ tumor cells/0.2 mL in female Swiss albino mice on day 28 of the experiment [34].

### 2.6. Behavioral Response Evaluation

They were kept in their cages for around 30 min in the testing room to get them accustomed to the new environment before they were tested. On the final day of the experiment, all behavioural tests were conducted in the same room. Behavioral analyses were applied as mentioned before in [10,35] and included the open field, inclined plane, tail suspension, posture, and swimming performance tests.

#### 2.6.1. Open Field Test

Behavioral measures were recorded for 5 min, commencing 2 min after the mice were placed in the test cages in the open field. In order to avoid any possible cueing effects from prior animals’ scents, the open field equipment was thoroughly cleaned with 5% ethanol. According to this study, the number of floor sections approached with two feet, the number of times the animal stood on its hind legs, stereotypical counts (the amount of grooming actions), and immobility duration (freezing) were measured (total time in second without spontaneous movements). To measure the level of fear in the open field, researchers counted the number of times mice entered a center square or region thought to be unprotected [36].

#### 2.6.2. Inclined Plane Test

This was calculated using an ordinary stretcher at close to a 5° incline, on which the mice were kept straight on a flat plane, with their head facing the side of the board that would be elevated; the complete method was described by Abou-Donia et al. [37] and Yonemori et al. [38]. The descent of thr mice was timed in order to determine the angle at which it began to slip backward, once this occured the trial was over. Results from the two trials, each separated by one hour, were averaged.

#### 2.6.3. Tail Suspension Test

During a 5-min period, researchers tracked how much time participants spent immobile. The tail suspension has been tested using the technique described [39]. In this test, mice were isolated by adhesive tape about 1 cm from the tail tip and suspended 60 cm above the floor. A plywood square platform was installed horizontally 15 to 20 cm under the bench to keep an eye on the platform. Only mice that were hanging motionless were considered immobile.

#### 2.6.4. The Postural Reflex Test

This test is mostly applied for estimating the sensorimotor function, it was done according to the method described by [40]. The test involves suspending mice 20 cm above the floor by their tails then assessing the degree of abnormal posture. A score of 0 was recorded for normal micethat stretched their forelimbs toward the ground. While a score of 1 is given to those with their forelimbs in unusual positions, such as twisting the contralateral limb to the body, circling the shoulder and contralateral limb, or both.

#### 2.6.5. Swimming Performance Test

The swimming performance test was applied by placing each animal in the middle of the glass aquarium and being observed for 5–10 s. The position of the nose and mouth on the surface of the water was used to score the swimming performance as follows: 0, 1 and 2 where head and nose are below the water surface, nose below the water surface, nose and top of the head at or above the water surface and ears below the surface; 3, is the same as score 2 except that the water line was at the mid-ear level; and 4, similar to score 3 but the difference is that the waterline was at the bottom of the ears [41].

### 2.7. Sampling

Researchers drew blood from each mouse’s orbital vessels (median canthus) for six hours following the exposure. Proinflammatory cytokines were measured in the serum by centrifugation at 3000 rpm for 15 min and 10 min, and then stored at −20 °C. Brain tissue samples were taken from the euthanized mice, cleaned with physiological saline, and sorted into three separate containers. One part was used for the comet assay to estimate the DNA damage, another part was homogenized for assessing neurotransmitter levels and oxidative stress indicators, and the last aprt was fixed in 10% neutral buffered formalin for histopathological and immunohistochemical studies.

### 2.8. Determination of the Levels of the Neurotransmitters

A solution containing 10^7 M^ ascorbic acid and 1.1 ^M^ perchloric acid with 20 ng of DHBA/mL of internal standard was used for brain tissue specimen homogenization to estimate neurotransmitter levels (serotonin, dopamine (DA), and GABA). Reverse-phase high-performance liquid chromatography with an electrochemical detector and a C-18 column accurately determined neurotransmitter levels. Acetylcholinesterase (AchE) was estimated in the homogenate by sandwich Elisa kits from Cusabio (Houston, TX, USA) (CSB-E17521m), according to the manufacture instructions. 

### 2.9. Assays of Antioxidant Enzymes and Oxidative Stress Indicators (MDA and PC) and Proinflammatory Cytokines

Malondialdehyde (MDA) was evaluated by the method previously documented by [42]. Furthermore, the concentration of protein carbonyl in the brain tissue was detected colorimetrically via the ELISA kit supplied by Abcam, USA (ab238536). An ELISA kit (Cat. No. MBS 267513) (My BioSource, San Diego, CA, USA) was used for the detection of 8-Hydroxyguanosine (8-OHdG) according to the manufacturer’s instructions. The antioxidant enzymes were estimated accordingly in the brain homogenate of all EAC mice of different treated and control groups, including SOD, CAT, and GSH via the ELISA kit from My BioSource, San Diego, CA, USA Cat. No. MBS728474, Cat. No. MBS161633 and MBS034842, respectively, following the manufacturer’s guidelines. The manufacturer’s instructions were used to estimate TNF-α CSB-E04741m, IL-10 CSB-E04594m, and IL-1β CSB-E08054m levels in the homogenate of brain tissue; all of the proinflammatory cytokines were detected at wavelength 450 nm.

### 2.10. Comet Assay

The comet assay was performed to assess DNA damage in the brain tissue of mice after 40 days of being treated with nicotine, *CV*, or both and were compared to the untreated control. The comet assay used in this study was performed under alkaline conditions on ordinary microscope slides, as illustrated previously by Singh et al. [43]. Homogenization was used to isolate nuclei, and slides were prepared. Electrophoresis was performed for 15 min at 25 V and approximately 250 mA at 4 °C in the dark. A total of 50 mL of 20 g/mL ethidium bromide was used to stain the slides. With the help of an Optika Axioscope fluorescence microscope(OPTIKA, Ponteranica (BG), Italy), approximately 100 nuclei per slide were examined and photographed at a magnification power 400.

### 2.11. Histopathological Evaluation

After euthanasia, mice were necropsied and representative tissue specimens from the brain of ten animals per group were collected following the guidelines of Ruehl-Fehlert et al. [44] and fixed in 10% neutral buffered formalin solution for 24 h. The fixed specimens were washed in distilled water, dehydrated in ethyl alcohol, cleared in HistoChoice^®^ (Sigma-Aldrich, St. Louis, MO, USA)clearing agent, impregnated, and embedded in a paraffin-beeswax mixture (90% paraffin and 10% beeswax), divided into sections 5 µm thick, stained with hematoxylin and eosin dyes [45], and examined by light microscope. Next, multiparametric quantitative lesion scoring was carried out following the method described by Mohamed et al. [46] with a few modifications. Concisely, for each animal, five nonduplicated randomly selected microscopic fields (40 objectives) (50 images per group) were captured using AmScope digital camera (United Scope LLC, Irvine, CA, USA) attached to a Nikon light microscope (Nikon Instruments Inc., New York, NY, USA). The next image analysis was performed where the neurons were showing histological alterations (pyknosis, necrosis, and vacuolation) to the total number of the neurons and the frequencies of other lesions (congestions, hemorrhages, gliosis, and neuropil microcavitation). The images were calculated subjectively, and the results were expressed as percentages (means ± SE). 

### 2.12. Bcl-2 and Caspase-3 Immunohistochemical Investigation

For each animal, two consecutive 5 μm thick cerebral tissue sections were prepared; the first was stained for Bcl-2 and the second was stained for caspase-3 antigens using the rabbit polyclonal anti-Bcl-2 primary antibody (ab196495) at a 1/100 dilution, the rabbit monoclonal recombinant anti-caspase-3 primary antibody [EPR18297] (ab184787) at 1/1000 dilution (Abcam Inc.), and the 3,3′-Diaminobenzidine (DAB) chromogen and hematoxylin counterstain following the avidin-biotin-peroxidase complex immunohistochemical technique [47]. Negative control sections were also prepared by using phosphate buffer saline as a substitute for the primary antibodies to demonstrate whether the immunohistochemical staining is specific and to avoid the non-specific reactions and false-positive results. For the quantitative assessment of the Bcl-2 and caspase-3 immunoexpression, five nonduplicated randomly selected microscopic fields (40 objectives) of the same size (210 μm × 280 μm) for each animal (50 images per marker per group) were captured. Next, image analysis was performed by calculating the percentages of the positively stained Bcl-2 and caspase-3 area fractions compared with the total areas of the images using the Java image processing program, ImageJ version 1.33 software (available online on https://imagej.nih.gov/ij/download/src/) (accessed on 9 December 2021) via the color deconvolution plugins. The results were expressed as mean + S.E. and simplified using an area fraction five-point scale as follows; negative to weak expression; less than 10%, mild expression; 10–25%, moderate expression; >25–50%, strong expression n; >50–75% and overexpression; >75%.

### 2.13. Statistical Analysis

Data were statistically analyzed using version 23 of the SPSS program (SPSS Inc., Chicago, IL, USA). The averages of different groups and standard deviations were calculated. The one-way ANOVA test was performed for comparison between the different groups (i.e., Control, *CV*, Nicotine, and Nicotine + *CV*) and for comparisons between means of groups, a post hoc Duncan multiple range (DMR) test was used. The means followed by the same letter in each column are not statistically different from each other at the 5% probability level (*p*-value at 0.05). (*p*-value at 0.05). In the behavioral tests, we utilized the Kruskal–Wallis test, which is a non-parametric option for a one-way ANOVA to compare between distinct Groups. Test interpretation: 

A null hypothesis (H0): The samples come from the same population (i.e., there is no difference between sample means or proportions “*p*-value greater than 0.05”).

Alternative hypothesis (Ha): The samples do not come from the same population (i.e., there is a difference between sample means or proportions “*p*-value less than 0.05”).

## 3. Results

### 3.1. The HPLC Analysis of CV Main Components

Total phenolics, Flavonoids, and Polysaccharides are detected as the main active ingredients in the *CV* as shown in Table S1 in Supplementary Materials with chromatograms of the standars and the samples. The most abundant compound of flavonoids are quercetine (12.39 µg/mL), hesperidin (11.69 µg/mL), 7-OH flavone (5.77 µg/mL), and rutin (4.69 µg/mL). The phenolic compounds ellagic (11.03 µg/mL), cinnamic (8.36 µg/mL), catechol (3.55 µg/mL), and chlorogenic (3.26 µg/mL) making up the highest concentration. Rhamnose (8.23 µg/mL) and glucuronic acid (5.26 µg/mL) are considered the chief poly saccharides detected in the *CV* algae, followed by mannose (2.96 µg/mL), and xylose(2.44 µg/mL). Figure S1 introduces the HPLC chromatograms of Total Phenolics, Flavonoids, and Polysaccharides of *CV* microalgae.

### 3.2. Behavioral Observations

As shown in Table 1, the administration of oral nicotine for 40 days (28 days pre and 12 days post-induction of EAC) in female Swiss mice obviously decreased ambulation frequency, rearing frequency, and grooming frequency but prolonged the freezing time compared to the control group. Throughout the experiment, the administration of *CV* with nicotine reversed the observed behavioral changes in the open field test markedly in the *CV*+ nicotine group compared with the nicotine-exposed group. The inclined plane and tail suspension test in the nicotine-exposed group had a decrease of 39.9% and 3.1 fold, respectively. The combination treatment with *CV* caused an increase in both observed behaviors in this group compared with the nicotine-exposed group. The data presented in Table 1 indicate the notable increase in swimming performance due to oral administration of nicotine by a 2.6 fold increase. Then, when we observed the data presented in the group exposed to *CV* and nicotine at the same time, the swimming performance was more reduced compared with that of the nicotine-exposed group.

### 3.3. Mortalities

During the experimental period (40 days, 28 days before the induction and 12 days post-induction), the mortalities were recorded, and the number of mortalities was recorded in Table 2. The mortalitie were at the the highest level 7 days after the induction of EAC in the nicotine group. The pattern was increased on the 11th and 12th day; the total number of mortalities recorded in the nicotine group was nine mice out of 20 (45%). The mortalities recorded in the combination group (Nicotine + *CV*) recorded the opposite pattern; it was at its highest on the 6th day of induction and reduced until the 12th-day post-induction. The total number of mortalities recorded in this group was 4/20 (20%). Mortalities were absent in the group-administered *CV* for 28 days pre-induction and 12 days post-induction. During the experiment, the control group recorded a single mortality on the 11th day of EAC induction.

### 3.4. Effect of Nicotine and/or CV on the Antioxidant Enzymes and Oxidative Stress Biomarkers in EAC Swiss Female Mice

Results presented in Table 2 demonstrated the alterations of the various antioxidant enzymes in the brain tissue of treated mice after 28 days pre-induction of ECA and 12 days post-induction. A significant reduction in the SOD, CAT, and GSH percentage in treated mice of 71.01%, 53.4%, and 73.6%, respectively, was observed in the nicotine EAC group compared with the control EAC group. These disturbances in the antioxidant’s activity were reduced in EAC-bearing mice that were administered *CV* orally in combination with nicotine, and this reduction was presented as a percentage of increase, which is estimated to be an increase of 60.09% in SOD, 53.82% for CAT, and a near two-fold increase in GSH regarding the nicotine-exposed group. Meanwhile, there was a significant (*p* < 0.001) increase of 1.65 and 5.35 fold in the levels of both MDA and PC, respectively, in the EAC-bearing mice that were orally administered nicotine solution in 2% saccharine by when compared to the control. This was reversed due to the administration of *CV*, which ameliorated the oxidative stress in the brain tissue of the nicotine + *CV* EAC-bearing mice group by 36.6% and 53.82%, respectively, compared with the nicotine group.

### 3.5. Effect of Nicotine Exposur, CV Exposure, or Both on Brain Neurotransmitter Levels

Table 3 displayed a significant increase (*p* < 0.001) of GABA and DA, (*p* < 0.001) after oral nicotine treatment in Swiss mice before and after the induction of EAC on of 12.16%, 2 fold, 4.36 fold, respectively, and an AChE increase of 50.37% when compared with the control. A significant reduction of 5.84%, 6.1%, 55.8%, and 29.3% for GABA, DA, serotonin, and AChE, respectively, was observed in the group concurrently exposed to nicotine and *CV*. When one examines the presented data in Table 3, one will find that the *CV* adjusted the levels of GABA, DA, serotonin, and AChE by 2.13 fold, 2.19 fold, 7.03 fold, and 4.2% in *CV*- treated group compared with obtained values in the control non-treated EAC-bearing mice.

### 3.6. Effect of Nicotine Exposure, CV Exposure, or Both on Inflammatory Markers

The female bearing-EAC Swiss mice that were administered nicotine for the 40 days revealed a significant *(p* < 0.001) increase in the levels of TNF-α, IL-1β of 1.3 and 1.9 fold, respectively, relative to the control group. However, oral nicotine exposure caused a substantial decline in the IL-10 levels in the serum of the treated group of 70.9% compared with the control group. Nevertheless, a significant *(p < 0.001)* reduction of 24.35% and 38.99 in the levels of TNF-α and IL-1β, respectively, were observed in the combined treated group (Nicotine + *CV*), relative to the nicotine-administered group. On the other hand, *CV* elevated a significant (*p* < 0.001) rise of 94.46% in the level of IL-10 when co-administered with nicotine compared with the nicotine group. 

### 3.7. Effect on DNA Damage (Comet Assay and 8-OHDG)

**Figure 1:**
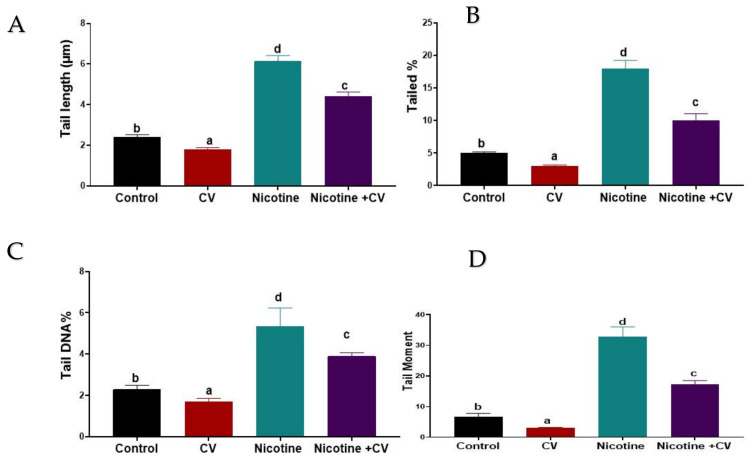
Effect of the oral administration of nicotine (100 µg/mL/kg bw), *CV* (100 mg/kg BW), or both for 40 days on the comet parameters in the brain tissue of female Swiss mice. (**A**): Tail length; (**B**): Tailed %; (**C**): Tail DNA %; (**D**): Tail moment; Data expressed as mean ± SE, *n* = 10 for each group. Each bar carrying different letters (a, b, c, and d) was significantly different at *p* < 0.001.

The comet assay was performed to assess DNA damage in the brain tissue of mice before and after EAC-induction for 40 days after treatment by nicotine, *CV*, or both was compared to the untreated control. The results of the comet assay are shown in Figure 1. A significant increase (*p* < 0.001) in DNA damage was indicated by an increase in tail length; the tail movement was greatly reduced compared with the control group. The detected fractions of the comet assay technique were mostly reversed in the brain tissue via oral administration of CV to the EAC-bearing mice at the end of the experiment. Notably, treatment with *CV* alone showed significantly lower DNA damage compared with the untreated control mice. Concerning changes in 8-OHdG levels and significant effects of nicotine solution-exposure (*p* < 0.001) were demonstrated by a 3.25 fold increase in the nicotine group compared to the control group. Meanwhile, the mice that were subjected to both *CV* solution concurrently with nicotine solution from the first to the last day of the experiment had 65.81% less oxidative DNA damage expressed by 8-OHdG level compared with the nicotine-exposed group.

### 3.8. Pathological Findings

**Figure 2:**
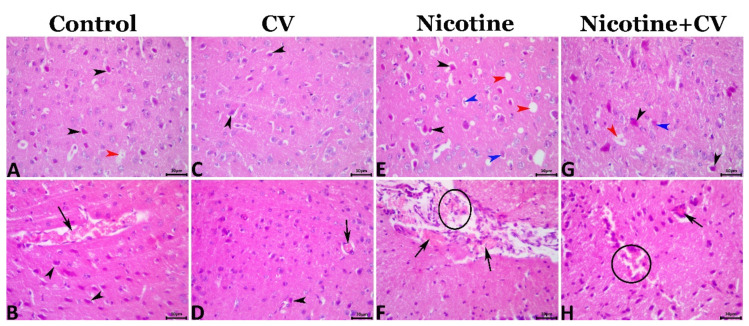
Representative photomicrograph of the cerebral tissue sections showing the immunoexpression of Bcl-2 (red arrowheads) in the control (**A**), *CV* (**B**), nicotine-treated (**C**), and nicotine + *CV*-treated (**D**) and the immunoexpression of the caspase 3 (red arrowheads) in the in the control (**E**), *CV* (**F**), nicotine-treated (**G**), and nicotine + *CV*-treated mice (**H**). Scale bar (30 μm).

The microscopic examination of the cerebral tissue sections showed various degrees of encephalopathy in different groups. Most of the cerebral tissue sections of the EAC mice (control mice) exhibited mild degenerative, necrotic, and circulatory alterations including shrunken neurons with hypereosinophilic cytoplasm and pyknotic nuclei, perinuclear vacuolation, neuropil microcavitation, and cerebral congestions (Figure 2A,B). A few sections showed glial clustering with or without neuronophagia. *CV* supplementation markedly rescues the cerebral histology from the EAC-associated encephalopathic alterations but did not maintain the normal cerebral histology as few degenerative and circulatory changes were evident in the *CV*-treated animals including neuronal pyknosis, perineural vacuolations, and cerebral congestions (Figure 2C,D). Exposure to nicotine significantly aggravated the EAC-associated encephalopathic histological changes, as a marked increase in the frequencies and severities of these alterations were noticed in nicotine-treated animals. Most cerebral sections exhibited neuronal pyknosis and necrosis, perineural vacuolation, neuronophagia, focal gliosis, neuropil cavitation, cerebral and meningeal congestions, and hemorrhages (Figure 2E,F). *CV* supplementation to the EAC-bearing-nicotine-treated animals significantly reduced the severity but not the frequency of the EAC-associated and nicotine-induced encephalopathic histological alterations in the nicotine + *CV*-treated animals. The most encountered lesions included neuronal shrinkage, necrosis neuropil microcavitation, and cerebral congestions (Figure 2G,H).

### 3.9. Immunohistochemical Findings

**Figure 3:**
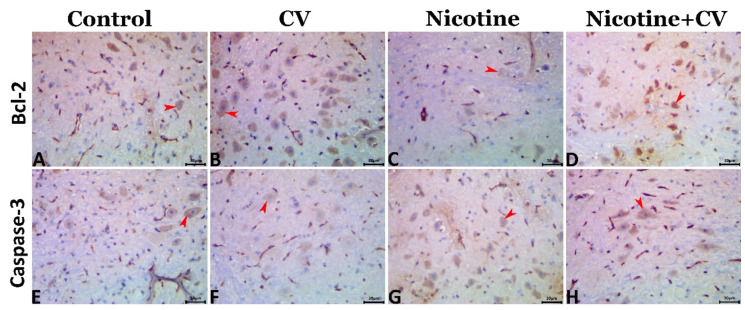
Representative photomicrograph of the cerebral tissue sections showing the immunoexpression of Bcl-2 (red arrowheads) in the control (**A**), *CV* (**B**), nicotine-treated (**C**), and nicotine + *CV*-treated (**D**) and the immunoexpression of the caspase 3 (red arrowheads) in the in the control (**E**), *CV* (**F**), nicotine-treated (**G**), and nicotine + *CV*-treated mice (**H**). Scale bar (30 μm).

The Bcl-2 and caspase 3 positively stained surface area fractions in all groups were shown in Figure 3A–H and were quantified in Table 4. The statistical data obtained from the image analysis emphasized that exposure to nicotine significantly downregulated the Bcl-2 and markedly upregulated the caspase 3 immunoexpression compared with the EAC-bearing animals. In contrast, *CV* supplementation in the *CV*-treated and nicotine + *CV*-treated animals significantly upregulated the Bcl-2 and downregulated the caspase 3 immunoexpressions compared with that in the EAC-bearing animals.

## 4. Discussion

Mood and anxiety disorders and schizophrenia are neuropsychiatric disorders that are mostly related to nicotine addiction [48,49,50,51,52]. There is still a lack of understanding on whether nicotine-linked toxic assault exacerbates the likelihood of behavioral health symptoms in later life, as various disease stressors, such as cancer, do. Many pre-clinical and clinical studies have found a strong correlation between nicotine and tobacco-containing compound habituation and increased anxiety symptoms mitigated by nicotine exposure [53]. The neurochemical changes evoked by nicotine exposure in the current study are accompanied by many behavioral alterations in the female mice after oral exposure to nicotine before and after the induction of EAC. Subsequent analysis reveals alterations in the anxiety-linked behaviors of the open field test, including ambulation frequency, rearing frequency, and grooming frequency, which expresses the activation of both motor functions and anxiety caused by the effect of nicotine. The same line of behavioral alterations exerted by chronic exposure to nicotine was previously demonstrated by Chris Ajonijebu et al. [54] and Royal et al. [55].

Moreover, nicotine caused a reduction in other behaviors, including the inclined plain test, tail suspension test, posture reflex test, and swimming performance test. All of these behavioral changes were most prevalent in the nicotine-exposed group throughout the experiment. Contradictory data were recorded by [53,56]. Conversely, Acri et al. [57] found that nicotine improved performance in various memory and learning tests, including working memory, spatial learning, and the passive avoidance task; rodents’ fear-associated learning as also observed in the Morris water maze. These behavioral changes observed in our study may occur due to the effect of nicotine on different brain regions, which is accompanied by brain cell loss [58]. Furthermore, previous records of long-term exposure to nicotine show that it caused alterations in cholinergic synaptic activity, especially in the hippocampus [59]. Many authors discovered that nicotine exposure induced changes in the learning of reward tasks in rats [59,60]. As a result of the nicotine treatment, several pathological changes in the brain tissues of female mice were discovered. These changes included clear signs of neuronal pyknosis and necrosis as well as vacuolation in the perineural space and neuropil cavitation. The former lesions may imply that frequent brain region aberrations are responsible for the sensorimotor inconsistencies expressed by anxiety and stimulated motor behaviors [35].

Accumulating evidence has confirmed that long-term exposure to nicotine boosts the excitatory mechanism of α7 nAChRs, which causes a glutamate release in the NAc (nucleus accumbens), PFC (prefrontal cortex), VTA (ventral tegmental area), and hippocampus [61,62,63]. Glutamate released in the dorsal striatum, VTA, and NAc, which is confirmed in several studies by microdialysis-based methods, have been enhanced by nicotine administration [64,65]. Herein, the oral administration of nicotine enhanced the production of DA and serotonin concentration in the brain tissue; this was previously evident in studies on the chronic nicotine systemic injection (0.4 mg/kg, Intraperitoneal (i.p). for 2 weeks) states of nicotine in which an elevated serotonin release was obvious in the STR in male Wistar rats and frontocortical area in male Sprague–Dawley rats [66,67]. GABAergic neuron activation and desensitization has been reported in acute conditions of nicotine exposure; the desensitization of GABAergic neurons c also be expected with chronic nicotine treatment, which implies that there will be little availability of GABA inhibitory neurotransmitters in the brain in the nicotine-treated group of EAC-bearing mice in the present experiment. The available literature assumes that chronic nicotine exposure alters the homeostasis of several neurotransmitters in the brain, which is thought to be the primary cause of nicotine dependence [68]. Nicotine affects dopaminergic neurons by activating and desensitizing nAChRs, which in turn increases excitability. Exogenously administered nicotine directly stimulates nAChRs, therefore, enhanced dopamine transmission is evident. Within the VTA (ventral tegmental area), predominantly α4β2-containing and α7 homomeric nAChRs were stimulated. On glutamatergic terminals, nicotine stimulates nAChRs that cause the release of an excitatory neurotransmitter (glutamate) GABA, an inhibitory neurotransmitter, which results in elevated dopamine release in the frontal cortex and nucleus accumbens. Nicotine also excites nAChRs on GABA-releasing terminals. Thus, nicotine increases the levels of GABA [69]. However, the relationship between the higher doses of nicotine and it being present for longer, the rapid desensitization of nAChRs on the GABA neuron, and the required α7nAChRs desensitization on the glutamate neuron result in a greater overall increase in dopamine levels as presented in the results of the in vivo testing in the current study.

**Figure 4:**
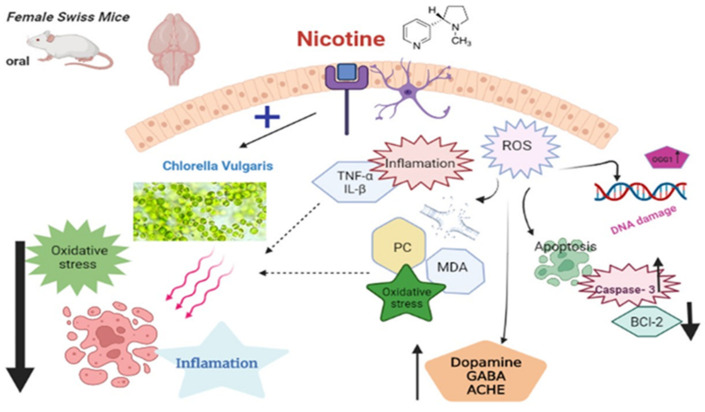
A diagram describing the mechanisms of nicotine-mediated neurobehavioral dysfunction and how or at what level does *Chlorella vulgaris* protect against the dysfunction.

To find out how damaging nicotine was to the brain of EAC-bearing mice, we estimated the levels of oxidative stress, apoptosis, and microscopical lesions in the brains of the mice. It was then propounded that oral exposure to nicotine caused elevation in the levels of MDA and PC while suppressing the activity of CAT, SOD, and the antiapoptotic marker Bcl-2 in the brain tissue. Moreover, the levels of immunohistochemical reactivity of caspase-3 and the elevation of DNA damage and proinflammatory cytokines in the brain tissue were also enhanced, and several lesions were observed during the examination of HE stained tissue sections. In vivo experiments in rats have shown that chronic nicotine administration causes a prooxidant/antioxidant status imbalance in their blood cells, plasma, and tissues [70]. Furthermore, DNA-damaged lymphocytes and imbalances in the prooxidant/antioxidant status were demonstrated in vitro with nicotine [71]. Peroxidation of the membrane lipids causing the formation of malondialdehyde (MDA) is the most observable sign of oxidative stress resulting from the generation of free radicals that induce several types of tissue damage [72]. It was previously observed that exposure to nicotine in cigarette smoke causes an increase in the levels of hydroperoxides, conjugated dienes, free fatty acids, and MDA [73,74]. The same pattern of decreased GPX and SOD enzyme activity was also found in the [75] study during nicotine exposure. Oxidative stress is one of the major underlying mechanisms in brain tissue damage and apoptosis. Enzyme activities that neutralize superoxide anions and hydrogen peroxide, which produce hydroxyl radicals responsible for a wide range of hazardous processes, could be impaired [10,76,77]. Consistent with our results, those obtained by Khanna et al. [78], show that the overexpression of chemokines and exposure to cigarette smoke in these animals can elicit numerous immunological and oxidative responses that may play crucial roles in the etiology of CNS inflammatory neurological disorders [79]. In addition, the available literature proved the ability of nicotine to produce copious amounts of ROS like NO and H_2_O_2_, which can act as signal-transducing agents for oxidative stress and inflammatory mediator activation in all experiments including cell cultures and animals [80]. The enhanced inflammatory response observed in the present study may have followed the mechanism of activating NF-kappaB. An inducible nuclear transcription factor identified in neurons, has since been implicated in a variety of biological processes including inflammation, innate immune response, cell development, apoptosis, and anti apoptosis and is activated by nicotine. Figure 4 illustrates the pathways through which nicotine could cause the brain tissue damage.

Neuroprotective substances with few or no adverse effects in comparison with currently available synthetic medications are being sought by researchers. The ability of algal cells to create various secondary metabolites, such as carotenoids, and their function in renewable fuel production, polyphenols, flavonoids, sterols, polyunsaturated fatty acids, and polysaccharides have recently attracted a lot of interest in microalgal research. Multiple pharmacological activities and neuroprotective potential are demonstrated by these compounds [81]. From the crucial components of *CV* are flavonoids, which have a variety of neuroprotective properties in the brain, including the ability to protect neurons against neurotoxic harm, to decrease neuroinflammation, and to improve memory, learning, and cognitive performance [82,83]. The hydroxyl groups on flavonoids, which are key plant ingredients, may give them an anti-oxidative effect by scavenging free radicals. Mutagenesis and carcinogenesis are inhibited in humans when up to 1 g of polyphenolic substances from a diet rich in fruits and vegetables is ingested daily [84]. The food industry is becoming more interested in phenolics because of their capacity to slow the oxidative degradation of lipids, which improves the quality and nutritional content of foods [85]. In plants, polysaccharides are made up of 100 or more monosaccharides. There are numerous therapeutic uses for these high molecular weight polymers as naturally occurring biological ingredients. These include anticancer, immunological modulation, anti-inflammatory, and antioxidant characteristics [86]. So the presence of all of these components, proven by HPLC analysis of the *CV* extract, may remove any outbursts about the obviously presented benefits of *CV* compared with any detectable nicotinic damage caused by oral exposure to nicotine in EAC-bearing mice. 

In the current study, to explore the protective effect of *CV* on the protection of the brain from the damaging role of nicotine, the concurrent administration of *CV* was applied during the exposure to nicotine in EAC-bearing mice. The results indicated that *CV* could reduce the number of mortalities caused by EAC induction and exposure to nicotine; the the highest mortality level was recoreded in the last 12 days of the experiment. Earlier investigations indicated that *CV* had immunostimulant characteristics, particularly in Ehrlich ascites tumor-induced myelosuppression, and that they extended the survival of rats by increasing the quantity and quality of phagocytes, primarily neutrophils, in chemotherapy-exposed tumor-bearing mice [31,87], alongside its efficient antitumor properties found in vitro in HeLa cancer cell lines according to El-Fayoumy et al., [88]. The positive and supportive role of *CV* in the brain tissue of Swiss mice is represented by elevated antioxidant enzymes activity with an antiapoptotic role. In addition, *CV* could markedly rescue the cerebral histology from the nicotine-EAC-associated encephalopathic alterations as previously recorded [88,89]. Oxidative stress-induced cell death could be delayed by using an extract of *CV*. Furthermore, it is found that DPPH radicals, for example, were scavenged by chlorella water extracts [27]. By increasing superoxide dismutase (SOD), catalase (CAT), glutathione peroxide (GPX), and glutathione (GSH) levels and decreasing malondialdehyde (MDA) levels, *CV* reduced lead-induced oxidative damage in rats’ brains [90,91]. *CV* contains a lot of bioactive phytochemicals and flavonoids which can exert several key roles in human and animal health.

As a result of its nutritional and medicinal properties, such as the elimination of free radicals and the reduction of blood lipids, β-1,3-glucan, one of the essential polysaccharides present in *CV* as presented in the results of chromatogram of our study, has been increasingly popular in recent years. [92,93]. Furthermore, *CV* is a wealthy source of ascorbic acid lutein, tocopherol α-, and β- carotene, which play a crucial role as neurotropic and stress relieving agents in many studies [94,95]. All of the properties mentioned above and the immense characters of *CV* can explain the recorded data from the present study. The concurrent administration of *CV* with nicotine in EAC-bearing mice stimulated the neurotropic functions, enhanced healthly brain activity, promoted the antioxidant status, reduced inflammatory signs, decreased mortalities, and limited the lesions evaluated by light microscopy. This observed improvement in the detected levels of neurotransmitters is also linked to the different bioactive components in *CV*, which were previously adopted in several studies for the screening of the phytochemical screening and antioxidant activity of *CV* [96,97].

## 5. Conclusions

The obtained results propose that, in the EAC-bearing mice, the brain is a target organ for the toxicity of oral nicotine exposure (100 µg/kg BW), which led to behavioral changes that may be correlated with continuous dopamine GABA secretion and inflammatory state activation. The damaged brain tissue may also result from the elevated apoptosis activating cascade reaction and DNA damaging effect of nicotine. The *CV*-supplemented brains of the EAC-bearing mice revealed activated antioxidant enzymes, which reduced the apoptotic and inflammatory potential of nicotine on the brains of the mice. Histopathological studies of brain samples after supplementation with *CV* has suggested regenerative changes and reduced apoptotic reactivity. Therefore, it seems likely that these changes will help protect the brain and regulate levels of neurotransmitters. *CV*’s enormous components, including polysaccharides, flavonoids, and carotenes, play a major role in *CV*’s protective properties. Clinical trials encourage cancer patients who are also exposed to nicotine to protect themselves from nicotine’s side effects. Further studies are required to influence the underlying mechanisms that could provide protection.

## Figures and Tables

**Table 1:** Open field, inclined plain, tail suspension, posture reflex, and swimmer performance tests following nicotine oral administration (100 µg/mL/kg BW for 40 days) and/or *C. vulgaris* in EAC Swiss female mice (*n* = 10).

Behavioral Tests		Experimental Groups				
		Control	*CV*	Nicotine	Nicotine + *CV*	*p*-Value
Open field test	Ambulation frequency	1.33 ± 0.88 ^c^	1.33 ± 0.33 ^c^	3.33 ± 0.33 ^a^	1.667 ± 0.33 ^b^	0.03
	Rearing frequency (anxiety-linked behaviour)	0.662 ± 0.58 ^c^	0.667 ± 0.33 ^c^	3.663 ± 0.33 ^a^	1.333 ± 0.33 ^b^	0.034
	Grooming frequency (anxiety-linked behaviour)	1.000 ± 0.01 ^c^	1.001 ± 0.01 ^c^	2.333 ± 0.33 ^a^	1.333 ± 0.33 ^b^	0.044
	Freezing time (Latency) (second)	1.000 ± 0.02 ^c^	0.667 ± 0.01 ^c^	3.333 ± 0.34 ^a^	1.667 ± 0.14 ^b^	0.046
Inclined plain test		46.667 ± 1.67 ^c^	48.333 ± 4.41 ^b^	45.33 ± 1.67 ^d^	50.667 ± 1.67 ^a^	0.034
Tail suspension test		10.333 ± 0.88 ^b^	10.333 ± 0.33 ^b^	9.514 ± 0.58 ^c^	11.000 ± 0.58 ^a^	0.027
Posture reflex test		0.000 ± 0.00	0.000 ± 0.00	1.000 ± 0.00 ^a^	0.333 ± 0.33 ^b^	0.041
Swimming performance test		0.000	0.000	0.667 ^b^	1.667 ^a^	0.023

Values are mean ± SE. Means within the same column are significantly different at *p* < 0.05. The different letters on the values ^a,b,c,d^ indicates the values are significantly different.

**Table 2:** Effect of oral administration of nicotine (100 µg/mL/kg BW) and/or *Chlorella vulgaris CV* (100 mg/kg BW) on the oxidative stress biomarkers in brain tissue of female Swiss mice (40 days) (*n* = 10).

Oxidative Stress	SOD (U/mL)	CAT (U/L)	GSH (mmol/L)	MDA (nmol/mL)	PC (ng/mL)	Mortality
Control	10.400 ± 1.16 ^a^	182.400 ± 5.26 ^a^	5.970 ± 0.68 ^a^	21.680 ± 1.22 ^c^	15.527 ± 0.35 ^c^	1/20
*CV*	11.340 ± 0.44 ^a^	193.333 ± 5.19 ^a^	6.890 ± 0.88 ^a^	19.157 ± 0.75 ^c^	12.967 ± 0.99 ^c^	0/20
Nicotine	3.017 ± 0.19 ^c^	84.900 ± 3.9 ^c^	1.573 ± 0.52 ^c^	57.607 ± 2.49 ^a^	130.600 ± 11.07 ^a^	9/20
Nicotine + *CV*	4.830 ± 0.57 ^b^	130.600 ± 11.07 ^b^	4.330 ± 0.52 ^b^	36.513 ± 0.62 ^b^	84.900 ± 3.9 ^b^	4/30

SOD: Superoxide dismutase; CAT: catalase; GSH: reduced glutathione; MDA: malondialdehyde; PC: protein carbonyl. Values are mean ± SE for ten samples/group. Means within the same column (in each parameter) carrying different superscript letters are significantly different at *p* < 0.001.

**Table 3:** Effect of the oral administration of nicotine (100 µg/mL/kg BW), CV (100 mg/kg BW), or both on the level of proinflammatory cytokines in the brain tissue and the concentration of 8-OHDG in the serum of female Swiss mice (40 days) (*n* = 10).

**Neurotransmitters in EAC**	**GABA (ng/mL)**	**DA(ng/mL)**	**SE (ng/mL)**	**AchE (mU/mL)**
Control	95.100 ± 7.13 ^c^	7.767 ± 0.76 ^c^	2.417 ± 0.49 ^c^	5.367 ± 0.56 ^c^
*CV*	92.467 ± 2.71 ^c^	7.017 ± 0.7 ^c^	2.587 ± 0.56 ^c^	4.215 ± 0.32 ^c^
Nicotine	106.667 ± 1.14 ^a^	23.267 ± 0.42 ^a^	12.977 ± 2.89 ^a^	8.067 ± 0.17 ^a^
Nicotine + *CV*	100.433 ± 2.21 ^bc^	13.333 ± 0.91 ^b^	5.790 ± 0.64 ^b^	5.741 ± 0.91 ^b^
**Inflammatory Response**	**TNFa (pg/mL)**	**IL-1B (pg/mL)**	**IL-10 (pg/mL)**	**8-OHDG g/mol**
Control	324.567 ± 6.01 ^c^	451.367 ± 3.417 ^c^	100.367 ± 0.86 ^a^	4.957 ± 0.45 ^c^
*CV*	322.700 ± 5.1 ^c^	377.148 ± 3.12 ^d^	103.467 ± 1.1 ^a^	4.837 ± 0.36 ^c^
Nicotine	750.767 ± 3.29 ^a^	1309.533 ± 4.57 ^a^	29.200 ± 5.05 ^c^	14.933 ± 0.32 ^a^
Nicotine + *CV*	567.900 ± 11.2 ^b^	799.100 ± 5.09 ^b^	56.833 ± 0.95 ^b^	8.167 ± 0.65 ^b^

GABA: gamma-Aminobutyric acid, DA: dopamine, SE: serotonin, AchE: acetyle choline esterase, TNFa: tumour necrosis factor α, IL-1B and IL-10: inter leukin 1B and 10, 8-OHDG: 8-hydroxy 2-deoxyguanosine.Values are mean ± SE for ten samples/group. Means within the same column (in each parameter) carrying different superscript letters are significantly different at *p* < 0.001.

**Table 4:** Effect of the oral administration of nicotine (100 µg/mL/kg bw), *CV* (100 mg/kg bw), or both on the concentration of Bcl-2, Caspase 3 immunoexpression, and encephalopathy lesion scoring of female Swiss mice brain.

Lesion	Control	*CV*	Nicotine	Nicotine + *CV*
Bcl-2 immunoexpression	30.179 ± 0.23 ^b^	59.469 ± 0.85 ^a^	13.702 ± 0.21 ^c^	30.320 ± 0.37 ^b^
Caspase 3 immunoexpression	7.964 ± 0.18 ^c^	0.979 ± 0.09 ^d^	37.794 ± 0.53 ^a^	13.109 ± 0.24 ^b^
Shrunken pyknotic neurons	6.809 ± 0.22 ^c^	3.659 ± 0.27 ^d^	24.021 ± 0.44 ^a^	13.702 ± 0.2 ^b^
Necrotic neurons	2.969 ± 0.36 ^b^	1.325 ± 0.16 ^b^	10.936 ± 0.66 ^a^	7.505 ± 0.42 ^a^
Perineural vacuolation	6.039 ± 0.2 ^c^	2.980 ± 0.22 ^d^	21.824 ± 0.33 ^a^	11.949 ± 0.18 ^b^
Neuropil microcavitation	10.400 ± 0.2 ^b^	2.300 ± 0.22 ^c^	13.100 ± 0.37 ^a^	8.700 ± 0.24 ^b^
Gliosis (astrocytosis and/or microgliosis)	1.400 ± 0.16 ^b^	0.600 ± 0.1 ^b^	3.600 ± 0.23 ^a^	1.000 ± 0.11 ^b^
Neuronophagia	0.600 ± 0.1 ^ab^	0.200 ± 0.06 ^b^	1.400 ± 0.13 ^a^	0.400 ± 0.08 ^b^
Cerebral congestion	2.600 ± 0.16 ^b^	1.200 ± 0.1 ^b^	7.400 ± 0.67 ^a^	3.600 ± 0.16 ^b^
Cerebral hemorrhage	0.600 ± 0.1 ^b^	0.200 ± 0.06 ^b^	2.200 ± 0.15 ^a^	1.000 ± 0.11 ^b^
Menengial congestion	0.600 ± 0.1 ^ab^	0.400 ± 0.08 ^b^	1.400 ± 0.1 ^a^	0.400 ± 0.08 ^b^
Meningeal hemorrhage	0.400 ± 0.08 ^a^	0.200 ± 0.06 ^a^	1.000 ± 0.11 ^a^	0.400 ± 0.08 ^a^
Bcl-2 immunoexpression	30.179 ± 0.23 ^b^	59.469 ± 0.85 ^a^	13.702 ± 0.21 ^c^	30.320 ± 0.37 ^b^

Values are mean ±SE for ten samples/group. Means within the same column (in each parameter) carrying different superscript letters are significantly different at *p* < 0.001.

## Data Availability

Not applicable.

## Supplementary Materials

The following are available online at https://www.mdpi.com/article/10.3390/biology11020279/s1, Figure S1: HPLC chromatograms of Total Phenolics, Flavonoids, and Polysaccharides of *CV* alga, Table S1: HPLC analysis of the main active components including (phenolic compounds, flavonoids and Polysaccharides) in *C.vulgaris.*
