# Supplementary material for: Sustained Functioning Impairments and Oxidative Stress with Neurobehavioral Dysfunction Associated with Oral Nicotine Exposure in the Brain of a Murine Model of Ehrlich Ascites Carcinoma: Modifying the Antioxidant Role of Chlorella vulgaris
Source: Biology (Basel). 2022 Feb 10;11(2):279. doi: 10.3390/biology11020279 (PMC8869302; doi:10.3390/biology11020279)
Supplement: Supplementary file 1 [file biology-11-00279-s001.zip › biology-1520617-supplementary.pdf]

Supplementary files

Table (S1) : HPLC analysis of the main active components including (phenolic compounds , flavonoids and Polysaccharides) in C.vulgaris

RT#	***Flavonoids***	***Concentration (µg/ml)***
3.2	7-OH flavone	5.77
4.5	Naringin	4.22
5.1	Rutin	4.69
7.0	Quercetin	12.39
8.0	Kaempferol	2.49
9.0	Luteolin	3.18
10.0	Hesperidin	11.69
12.0	Catechin	4.35
RT#	***Phenolics***	***Concentration (µg/ml)***
2.8	Chlorogenic	3.26
4.1	Catechol	3.55
5.1	Syringic acid	2.11
7.0	Cinnamic	8.36
8.0	Caffeic	2.87
9.0	Pyrogallol	0.69
9.8	Gallic	2.41
12.0	Salicylic	1.88
12.8	Ellagic	11.03
15.0	Benzoic	3.41
**RT#**	**Compound**	**Concentration (µg/ml)**
6.0	Rhamnose	8.23
8.0	Glucuronic acid	5.26
12.2	Mannose	2.96
15.0	Xylose	2.44

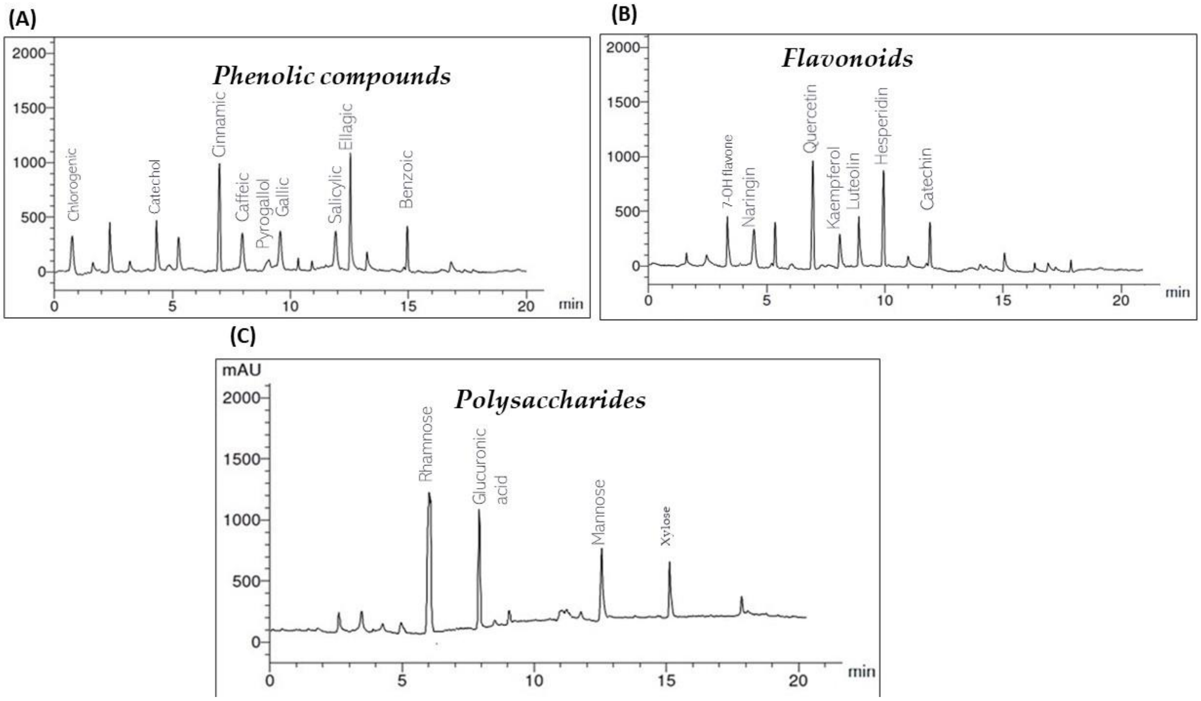

Figure (S1): HPLC chromatograms of Total Phenolics, Flavonoids and Polysaccharides of *CV* alga.
